# Extracting Free

**Published:** 1885-07

**Authors:** 


### Extracting Free.

A PARODY.

Read before the Mad River Dental Society.

The shades of night were falling fast,
As through a Buckeye village passed
A youth who showed to curious eyes
A banner with the strange device,
Extracting free.

His kit he ope'd with bated breath,
A turnkey, flashed from out its sheath,
And like a railway dinner-gong rung
The accents of that hated tongue,
Extracting free.

In the dentist's office on the right,
He caught a gleam of hate and spite,
For the dentist he did fume and growl
As, twixt his teeth there 'scaped a howl,
Extracting free.

"Try not this folly!" the old man said,
For if you do, you'll rob me of my bread,
The world is great and large and wide,
So get thee hence, here let me bide,
Extracting free.

"Oh stay" the dentist's daughter said,
Jack Shepard's shape had turned her head,
"And wait until the gums are healed,"
"Ah no," he cried, "their fate is sealed,"
Extracting free.

"Take heed, beware the dreadful dental law,
To punish such as break a jaw;"
This was the dentist's last good night
As Jack packed up and vanished out of sight,
Extracting free.

But hark! next morn is heard a novel sound,
Jack's partner, with the wagon coming 'round,
To put in place the store-teeth grand,
Advertised by Jack and his brass band,
Extracting free.

A victim, by the resident dentist was found,
With dyspepsia gaunt, his bowels bound,
But grasping still within his maw
The teeth that caused death by dread lock jaw,
Extracting free.

Quick hied the dentist to his Association,
To warn all patrons in this glorious nation,
'Twere better far to pay a good fee
Than die a sad death from
Extracting free.

